# Functional–structural root-system model validation using a soil MRI experiment

**DOI:** 10.1093/jxb/erz060

**Published:** 2019-02-19

**Authors:** Axelle Koch, Félicien Meunier, Jan Vanderborght, Sarah Garré, Andreas Pohlmeier, Mathieu Javaux

**Affiliations:** 1 Earth and Life Institute – Environmental Sciences, UCLouvain, Louvain-la-Neuve, Belgium; 2 Computational and Applied Vegetation Ecology Lab, Ghent University, Ghent, Belgium; 3 Institute of Bio- and Geosciences, IBG-3 Agrosphere, Forschungszentrum Jülich GmbH, Jülich, Germany; 4 Earth and Environmental Sciences, KU Leuven, Celestijnenlaan, Leuven, Belgium; 5 Gembloux Agro-Bio Tech, Université de Liège, Passage des déportés, Gembloux, Belgium

**Keywords:** Functional–structural root-system model, magnetic resonance imaging, root hydraulic conductivities, root water uptake, R-SMWS, tracer experiment

## Abstract

Functional–structural root-system models simulate the relations between root-system architectural and hydraulic properties, and the spatio-temporal distributions of water and solutes in the root zone. Such models may help identify optimal plant properties for breeding and contribute to increased water-use efficiency. However, it must first be demonstrated that they accurately reproduce the processes they intend to describe. This is challenging because the flow and transport processes towards individual roots are hard to observe. In this study, we demonstrate how this problem can be addressed by combining co-registered root and tracer distributions obtained from magnetic resonance imaging with a root-system model in an inverse modeling scheme. The main features in the tracer distributions were well reproduced by the model using realistic root hydraulic parameters. By combining the functional–structural root-system model with 4D tracer observations, we were able to quantify the water uptake distribution of a growing root system. We determined that 76% of the transpiration was extracted through 3rd-order roots. The simulations also demonstrated that accurate water uptake distribution cannot be directly derived either from observations of tracer accumulation or from water depletion. However, detailed tracer experiments combined with process-based models help decipher mechanisms underlying root water uptake.

## Teaser

For the first time, a functional–structural root-system model is validated by combining a tracer experiment monitored with magnetic resonance imaging and three-dimensional modeling of water and solute transport.

## Introduction

Functional–structural root-system models (FSRSM, see Meunier *et al.*, 2017; Ndour *et al.*, 2017) have been developed since the late 1980s (Diggle, 1988; Doussan *et al.*, 2006; Javaux *et al.*, 2008; Schnepf *et al.*, 2018). These models combine root functional and structural information to describe local processes in the soil–root continuum (Passot *et al.*, 2019). They aim to give us a better understanding of the relationships between root architecture, root development, hydraulics, and water flow and solute transport in the root zone.

Resolving processes around roots indeed helps us to understand, amongst other things, the development of the root hydraulic architecture (Zarebanadkouki *et al.*, 2016), the impact of salinity stress (Schröder *et al.*, 2013; Jorda *et al.*, 2018), the fate of pesticides in the root zone, the uptake of nutrients (Dunbabin *et al.*, 2004; Leitner *et al.*, 2010), the strategies developed by plants competing for resource acquisition (Postma and Lynch, 2012; Li *et al.*, 2015, 2016; Strock *et al.*, 2018), the interactions between soil structure and root growth (Landl *et al.*, 2016), and the impact of mucilage in root water uptake (Schwartz *et al.*, 2016). Moreover, these models can be used for optimizing root traits and to develop crop ideotypes (Leitner *et al.*, 2014; Meunier *et al.*, 2016).

However, the accuracy of FSRSMs to predict root water uptake (RWU) of a complex root system has so far never been validated with four-dimensional (4D; time and space) experimental data. Schröder *et al.* (2013) proved the ability of R-SWMS (Javaux *et al.*, 2008) to predict steady-state salt accumulation around a single root but not around an actual growing root system. Koebernick *et al.* (2015) simulated water uptake with a FSRSM using 4D root-system architectures (RSAs) that were derived from computed tomography measurements; however, the measured soil variables (i.e. the soil water potential) that were compared with simulation results were not spatially resolved around single roots but instead represented bulk soil measurements. Zarebanadkouki *et al.* (2016) did obtain spatially resolved information about the RSA and the water flow into roots from imaging the movement of deuterated water with neutron radiography; however, their techniques are limited to 3D experiments (2D space and time).

Experimental data for RWU and root hydraulic properties are required for a direct validation of models, and are very challenging to obtain. On one hand, measuring the magnitude and the spatial distribution of RWU remains complex and tedious despite the technical progress achieved in past decades to directly observe water movement in the soil–plant–atmosphere continuum (SPAC; Ahmed *et al.*, 2015). On the other hand, while being acknowledged as critical for plant performance (Leitner *et al.*, 2014), root hydraulic properties remain difficult to characterize for several reasons (Vadez, 2014). First, their determination is highly time-consuming (Li and Liu, 2010), and hence it is difficult to repeat them over the entire set of root ages, orders, and development stages. Second, while it has been demonstrated that these properties change dramatically with root type and age (Vetterlein and Doussan, 2016), and can be affected by environmental constraints (Hachez *et al.*, 2012), current experimental techniques are rather local. As a consequence, there is currently no agreement on a standard methodology to measure the distribution of root hydraulic conductivities in a whole root system (Rieger and Litvin, 1999; Meunier *et al.*, 2018b).

This lack of information on RWU distribution and on root hydraulic properties hinders the validation of FSRSMs in a direct way. However, an indirect validation remains possible by combining simulations and observations in an inverse modeling framework. In inverse modeling, the modeled outputs are compared to the experimental results and the error between them is calculated. This is repeated with varying model parameters until a minimal error or an error lower than a previously defined threshold value is reached. In other words, inverse modeling allows the derivation of information, from the outputs, on the model input parameters. In the case of a process-based model, it is assumed that the key processes are accurately simulated by the model. Inversion leading to a physically (or physiologically) based set of parameters therefore indirectly validates the FSRSM that is being used.

An accurate FSRSM validation through inverse modeling, while possible, requires important considerations in the experimental set-up. First, three- to four-dimensional (3D space with or without time) RSA is required. Nowadays, various non-invasive techniques allow RSA acquisition, such as magnetic resonance imaging (MRI; Stingaciu *et al.*, 2013), X-ray computed tomography (X-ray CT; Mairhofer *et al.*, 2012; Koebernick *et al.*, 2014), neutron computed tomography (neutron CT; Moradi *et al.*, 2011), or the combination of RGB and hyperspectral imaging (Bodner *et al.*, 2017). Information about root-zone processes (e.g. water content, water potential, or tracer dynamics) is then needed. Spatio-temporal distributions of water content can be obtained using X-ray CT (Hainsworth and Aylmore, 1983), neutron CT (Carminati *et al.*, 2010; Esser *et al.*, 2010; Tötzke *et al.*, 2017), and MRI (Pohlmeier, 2010). But water-content patterns do not contain a lot of information about the actual distribution of the water fluxes and local uptake rates (Vandoorne *et al.*, 2012). Imaging tracer distributions could be an alternative to indirectly derive distributions of water fluxes and could therefore be used to validate FSRSMs. Zarebanadkouki *et al.* (2012, 2013, 2014) and Tötzke *et al.* (2017) tracked deuterated water transport through the SPAC using neutron radiography and were able to quantify local RWU. More recently, MRI has been used to track the movement of gadolinium diethylene-triamine-penta-acetate in a sandy soil column (Haber-Pohlmeier *et al.*, 2017), offering the opportunity to determine when and where (i.e. by which roots) water is actually taken up.

In this study, we aimed to numerically reproduce tracer movement in a sand container planted with *Lupinus albus* in a system designed by Haber-Pohlmeier *et al.* (2017). We investigated how the 4D monitoring of the tracer in combination with process-based modeling could inform us about RWU dynamics and plant hydraulic properties. In particular, we focused on inverse modeling of the tracer distribution and accumulation in order to obtain the hydraulic properties of the root system using a physically based model of the water flow in the SPAC. The objective of this work was therefore three-fold. We aimed to (i) validate a FSRSM for water flow and solute transport in the root zone, (ii) determine how informative the evolution of a tracer distribution is for the RWU dynamics of a growing root system, and (iii) determine the most likely distribution of root hydraulic properties.

## Materials and methods

### Experimental set-up

We briefly summarize the experimental set-up here: for more detailed information, see Haber-Pohlmeier *et al.* (2017). A 7-d tracer experiment was performed in a column planted with white lupin (*Lupinus albus* L.), and root development, water content, and tracer concentration distributions were monitored over time using MRI.

**Fig. 1.**
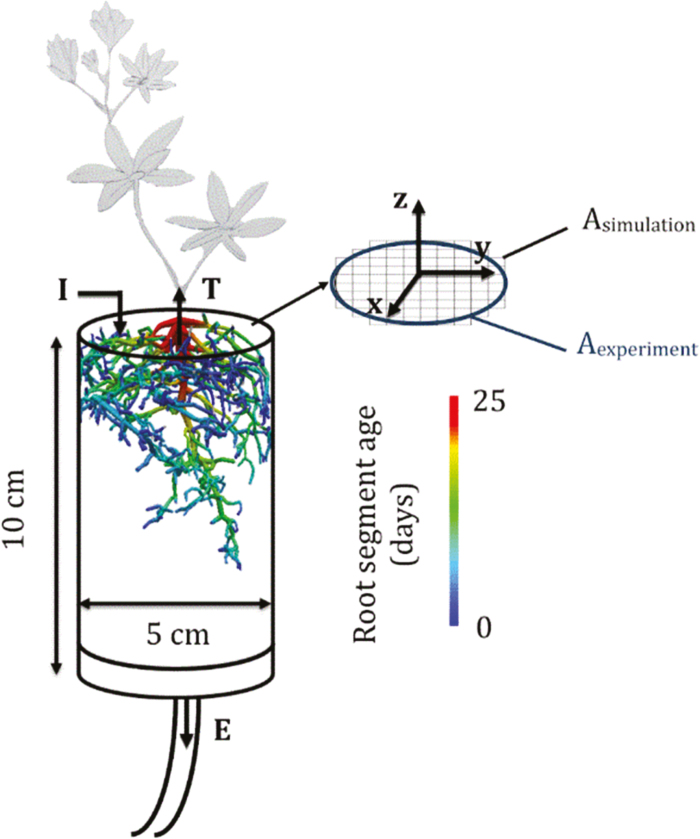
Diagram of the experimental set-up. The experimental column and the reconstructed final root architecture at 25 d after sowing are shown. The fluxes considered in the modeling are irrigation (I), transpiration (T), and effluent (E). The experimental and simulated column cross-sections are also shown.

After seed germination, a white lupin plant was grown for 18 d in a cylindrical column (10 cm high; 5 cm inner diameter; see Fig. 1) filled with sand (FH31, Quarzwerke Frechen GmbH, Frechen, Germany) under a 1/12 h day/night cycle at 60% relative humidity. The cylindrical shape of the column and the use of sandy medium are constraints linked to MRI technology. The tracer experiment started at 18 d after sowing (DAS; see Supplementary Fig. S1 at *JXB* online) and we used gadolinium diethylene-triamine-penta-acetate (Gd-DTPA^2−^), which is an MRI contrast agent that can be used as a tracer for solute transport in porous media thanks to its chemical inertness, its conservative transport properties, and its anionic net charge in neutral aqueous solution that prevents its adsorption on soil mineral surfaces (Haber-Pohlmeier *et al.*, 2010). The initial volumetric soil water content (*θ*; see list of variables, Table 1) that was imaged was 0.35 (cm^3^ cm^−3^). During the first 6 d of the experiment (18–24 DAS), the soil column was located under artificial lights (PAR sufficient for the plant to transpire and grow) and irrigated continuously with a 1 mmol l^−1^ Gd-DTPA^2−^ solution, except for the MRI scanning periods (~6 h d^–1^). The plant was not transpiring and was not watered during the scanning periods, which were performed overnight. During the last day of the experiment (24–25 DAS), irrigation was stopped so that the tracer and the water could redistribute in the sand substrate. The main driver of water movement in the column was then the plant transpiration. The cumulative fluxes of the irrigation solution, transpired water, and effluent together with the mean column water content are detailed in Supplementary Table S1 as they were observed or calculated (adapted from Haber-Pohlmeier *et al.*, 2017). The soil–root system was imaged daily during the entire experiment in order to obtain tracer and root distribution maps. However, due to technical problems with the scanner, no image was obtained the 6th day of the experiment (24 DAS). The experiment protocol and timing are summarized in Supplementary Fig. S1.

**Table 1.** List of variables

Name	Symbol	Units	Description
Root axial conductance	*K*_x_	cm^4^ hPa^−1^ d^−1^	Root segment capacity to transport water axially
Root radial conductivity	*k*_r_	cm hPa^−1^ d^−1^	Root segment capacity to transport water radially
Root system total hydraulic conductance	*K*_rs_	m^3^ MPa^−1^ s^−1^	
Root system total hydraulic conductivity	*k*_rs_	m MPa^−1^ s^−1^	*K*_rs_ normalized by the total root surface area
Water uptake density	WUD	cm^3^ d^−1^ cm^−3^	The volumetric flow of uptake per soil voxel volume
Water depletion	WD	cm^3^ cm^−3^	The water content change over successive observation times
Volumetric soil water content	*θ*	cm^3^ cm^−3^	
Soil water potential	*h*	MPa	
Soil bulk density	*ρ*_b_	g cm^−3^	
Tracer concentration	[Gd-DTPA^2−^]	mmol L^−1^	
Tracer accumulation	Acc.	mmol L^−1^	The increase of Gd-DTPA^2−^ concentration over the considered period
Solute longitudinal dispersivity	*α*_L_	cm	
Solute lateral dispersivity	*α*_T_	cm	

The scanner used for imaging the RSA and the tracer concentration in the liquid phase was a 1.5 T split-coil MRI scanner (Agilent Technologies) comprising a 300 mT m^–1^ gradient system and a 10-cm solenoid transmitter–receiver coil. The RSA was imaged using a T_2_ (the transverse relaxation time) weighted 3D fast-spin echo imaging sequence with a matrix size of 256×256×64 points for a field of view (FOV) of 60×60×70.4 mm^3^. The FOV started from the top of the soil column. The maps of Gd-DTPA^2−^ concentration in the liquid phase were acquired from 2D multislice spin echo sequences with inversion recovery preparation. The axial FOV was 60×60 mm^2^ with a matrix size of 256×256 points, and 40 axial slices of thickness 2 mm and gap 0.2 mm were imaged. The resulting spatial resolution of the voxels was 0.234×0.234×2.2 mm^3^. Both matrices (RSA and Gd-DTPA^2−^ concentration) were concentric. This means that the upper slice of the RSA matrix corresponded to the fifth slice of the Gd-DTPA^2−^ concentration maps.

### Reconstruction of RSA

The reconstruction of the RSA was based on previously described MRI measurements conducted by Haber-Pohlmeier *et al.* (2017). First, the MRI data were processed and segmented into binary images. We then reconstructed the RSA manually and extracted the root segment network. This was achieved on a Holobench, a 3D virtual reality system that runs on VISTA-Software (VISTAWurzel) (Wienke, 2009). We used 3D-polarized glasses and a 3D-computer mouse to track each root branch 3D location and radius from the binary images (Stingaciu *et al.*, 2013; Koebernick *et al.*, 2015). It should be noted that the MRI could not detect roots that had a diameter smaller than 200 μm.

To characterize the growing RSA, we started the reconstruction with the root system of the last day (MRI image taken at 25 DAS) and continued in inverse chronological order (MRI images taken at 23, 22, 21, 20, 19, and 18 DAS) by removing the last created root nodes step by step. After that, root origination time and branching order were linearly interpolated between successive images with MATLAB routines.

### Coupling of models and set-up

R-SWMS (Javaux *et al.*, 2008), a model that computes soil water flow based on the Richards’ equation and 3D water flow in the root system based on an explicit consideration of water potential gradients, was used to simulate the experiment. For the representation of tracer transport, R-SWMS was coupled with ParTrace (Schröder *et al.*, 2012), which solves the convection–dispersion equation based on a Lagrangian approach (random-walk particle tracking). R-SWMS provides ParTrace with the water content distribution as well as the velocity field, with which ParTrace simulates the particle movements and calculates the concentration distributions.

Since the mesh in R-SWMS is composed of cubic voxels, representing a cylinder would require an infinite number of voxels. A mesh of cubic 0.25×0.25×0.25 cm^3^ voxels was the chosen compromise between computational power and accurate representation of the cylindrical shape. Given the square shape of the grid elements, the simulated column surface area was slightly different from the one of the experimental column (Fig. 1). Therefore, the simulated input, output, and transpiration flows were adapted with the following weighting factor:

$$W_{\text{simulation}}=\frac{A_{\text{simulation}}}{A_{\text{experiment}}}\;W_{\text{experiment}}$$ (1)

where *W* is the water flows (irrigation, effluent, or transpiration; see Fig. 1) and *A* is the soil column surface. For a voxel size of 0.25 cm, the ratio between simulated and experimental surfaces (*A*_simulation_/*A*_experiment_) is 1.1. In addition, in the diagram in Fig. 1 we indicate the axis convention for R-SWMS. The vertical axis (*z*) is positive upwards, and we use ‘*xy*-averaged’ and ‘horizontally averaged’ to mean the same thing.

Soil hydraulic properties were modeled using the closed-form equations of van Genuchten–Mualem (Mualem, 1976; van Genuchten, 1980) and these functions were parameterized according to Schröder (2014), who worked with the same soil (see Supplementary Table S2 for parameters). Soil bulk density (*ρ*_b_) was set in the model to 1.62 g cm^−3^, its measured value. Regarding the solute transport parameters, the diffusivity of Gd-DTPA^2−^ was fixed to 0.35 cm^2^ d^−1^, as in Bechtold *et al.* (2011). The longitudinal (*α*_L_) and lateral (*α*_T_) dispersivities were set to 0.25 cm and 0.025 cm, respectively (the longitudinal value being set to its maximal theoretical value, the voxel width, 0.25 cm). Solute was considered not to be taken up by roots (i.e. exclusion), and an analysis of Gd-DTPA^2−^ mass balance at the end of the experiment showed that this assumption was true (Haber-Pohlmeier *et al.*, 2017).

Root growth and ageing were explicitly simulated using linear interpolations between successive MRI root system scans as explained above.

### Analyses

#### Determining optimal root hydraulic properties.

Two hydraulic parameters were defined for each single root segment: the radial conductivity (*k*_r_) and the axial conductance (*K*_x_). These properties depend on the root order and the segment age (Doussan *et al.*, 2006; Zarebanadkouki *et al.*, 2016). To avoid having an excessively large set of possible root architectures, we constrained their distributions according to several assumptions. First, we assumed that *k*_r_ and *K*_x_ were constant along the taproot, as in Zarebanadkouki *et al.* (2016). For the laterals (root orders 2 and 3), we assumed that the root segments younger than 5 d old had different hydraulic parameters than older root segments. The stepwise response of RWU to the root development processes supports that assumption (Vetterlein and Doussan, 2016). Furthermore, *k*_r_ of the older segments had to be smaller than that of younger ones. Indeed, the formation of the casparian band, which hinders water following the apoplastic pathway from the cortex to the stele, increases the root resistance to radial flow (Vetterlein and Doussan, 2016). In what follows, the hydraulic properties of the taproot will be identified by the subscript T and those of the lateral roots by the subscripts Ly for the young (0–5 d old) and Lo for the old (5–25 d old) segments. The 5-d-old threshold was chosen to give a similar root length for both age classes. The sets of tested radial conductivities and axial conductances were geometric sequences in the intervals [10^−5^ 10^2^] (cm hPa^−1^ d^−1^) and [10^−4^ 10^2^] (cm^4^ hPa^−1^ d^−1^), respectively. The resulting simulated Gd-DTPA^2−^ concentration maps, corresponding to the 15750 generated scenarios differing only in their root hydraulics parameterization, were compared to the experimental distribution.

To assess the fitness of a certain root hydraulic architecture to describe the observed tracer concentration distribution, the root mean-square of the error (RMSE) between simulated and observed spatial tracer concentration maps was used, and calculated as:

$$\text{RMSE}=\sqrt{\frac{\sum_{i=1}^{N}{\left({\left[Gd-DTPA^{2-}\right]}_{\text{obs},i}-{\left[Gd-DTPA^{2-}\right]}_{\text{mod},i}\right)}^{2}}{N}}$$ (2)

where [Gd-DTPA^2−^]_obs,*i*_ and [Gd-DTPA^2−^]_mod,*i*_ are the observed and modeled concentrations in voxel *i*, respectively, and *N* is the total number of observed voxels. Since the spatial resolution differed between the observed and modeled [Gd-DTPA^2−^] maps, the MRI results were averaged to match the simulated grid.

The simulated Gd-DTPA^2−^ maps were considered at the time corresponding to the end of the MRI scanning. Only the last day of the irrigation treatment and the non-leaching phase were simulated for the parameter optimization. Indeed, water movement (and hence tracer transport) mainly depended on soil characteristics during the leaching period (between 18–24 DAS), whereas it depended more on root properties during the non-leaching period (24–25 DAS), throughout which plant transpiration was the main driver of water movement. However, since the Gd-DTPA^2−^ distribution map at 24 DAS was not available (as the MRI facility did not work that particular day), we started the simulation at 23 DAS. Initial conditions (soil water content distribution, solute distribution, and root architecture at 23 DAS) and boundary conditions (irrigation, transpiration, and effluent flows) were defined according to the experimental conditions. As mentioned earlier, the Gd-DTPA^2−^ concentration maps obtained by MRI did not cover the entire soil column. Therefore, to generate the initial concentration distribution file, we calculated the quantity of Gd-DTPA^2−^ contained in the entire soil volume (difference between the amount of tracer present in the cumulative irrigated solution and in the cumulative effluent), we determined how much Gd-DTPA^2−^ was located in the part of the soil column that was imaged by MRI, and we added the difference in the non-monitored soil area. The tracer concentration was assumed to be uniformly distributed within the *xy*-layer and to linearly decrease with depth (see Results).

In R-SWMS, the roots do not occupy any explicit volume and so Gd-DTPA^2−^ could accumulate exactly where the water is taken up (at root nodes) whereas, in reality, the accumulation occurs around the root boundaries. This may introduce an overestimation of the actual Gd-DTPA^2−^ concentration if the model spatial resolution is too fine. Indeed, when considering voxels of 0.25 cm width, root segments occupied on average 1/2, 1/4, or 1/5 of the voxel volume for orders 1, 2, and 3 respectively. For this reason, the comparison was computed for merged voxels (0.5 cm width).

The best (i.e. leading to the lowest RMSE) parameter set was further locally optimized by exploring the parameter space with smaller and smaller ranges around the minimum until convergence.

#### Experiment sensitivity to root hydraulic properties

To determine the information content of the MRI experiment, a local sensitivity analysis was performed around the global optimum to assess which of the root hydraulic parameters the dataset was sensitive to. To do this, 1215 parameter sets were generated by systematically sampling 2D parameter cross-sections of the parameter space around the global optimum. The parameter domain was fixed in a range corresponding to 1/8 to 8 times the optimal parameters. The 3D tracer distribution sensitivity to root hydraulic properties was checked using the RMSE between the observed and the simulated values (eqn 2).

#### Evaluating proxies for water uptake

The model was then run over the entire experimental period (i.e. between 18–25 DAS) with the optimized parameters and with the same initial and boundary conditions as the experiment. This allowed us to determine the water uptake density (WUD) distribution and dynamics. WUD is the volumetric flow of uptake per soil voxel volume (cm^3^ of water d^−1^ cm^−3^ of soil). Since the tracer is supposed to be inert and not extracted by plant roots, solute should preferentially accumulate where the plant extracts soil water. If that hypothesis is confirmed, then the solute distribution map, obtained by MRI, informs us about cumulative WUD distribution. We also focused on determining what relationships existed between water depletion (WD, i.e. water content change over successive observation times) and WUD. The relationship between these variables and WUD was tested for the non-leaching period (between 24–25 DAS). All the variables were normalized using the following equation.

$${\hat{Y}}_{i}=\frac{Y_{i}}{\sum_{j=1}^{N}Y_{j}}$$ (3)

where *Y* is the variable of interest (Gd-DTPA^2−^ accumulation, WD, and cumulative WUD) and *Ŷ* is the corresponding normalized variable. Gd-DTPA^2−^ accumulation is defined as the increase of Gd-DTPA^2−^ concentration over the considered period and the cumulative water uptake density represents the total amount of water taken up by roots in each voxel over a certain time period.

## Results

### Root system architecture and development

The fully-grown (25 DAS) root system generated from the MRI scans is represented in Fig. 1 together with the age distribution of the root segments that were obtained. The total root length was 3.23 m, which corresponded to a mean root growth of 0.17 cm d^−1^ (taking into account the respective time of appearance of each root tip). This total root length refers to root segments with a diameter >200 μm, i.e. that could be detected by MRI. It appeared that roots were mainly located in the upper part of the soil column (79% of the total root length was found between 0–3 cm depth). Three root orders could be identified (1, taproot; 2, lateral roots connected to the taproot; and 3, secondary laterals) and contrasted in their contribution to the total root-system length: 2.4%, 27.6%, and 70% for root orders 1, 2, and 3, respectively. Root order had no significant impact on the elongation rate of orders 2 and 3 (means 0.16 cm d^−1^ and 0.17 cm d^−1^, respectively), while that of the taproot was higher (0.43 cm d^−1^). The mean root diameters were significantly different between the orders, being 0.045 cm for order 3, 0.06 cm for order 2, and 0.11 cm for the taproot. This difference between the taproot and laterals justifies the chosen scheme of hydraulic properties, namely that *K*_x_ and *k*_r_ differ between the taproot and the laterals of all orders (see Methods). The mean root lengths varied between 0.8 cm, 2.4 cm, and 7.7 cm for the 3rd, 2nd, and 1st root orders, respectively. On average, the lateral root density was 4.8 laterals cm^–1^ of taproot and 3.3 second-order laterals cm^–1^ of lateral root.

### Simulated versus observed tracer distributions

**Fig. 2.**
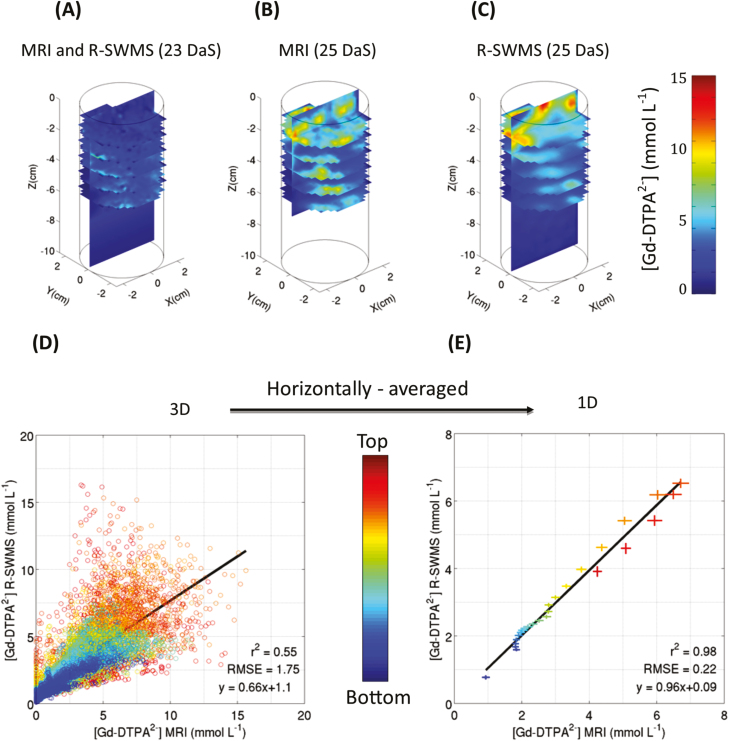
(A) 3D concentration distribution of the Gd-DTPA^2−^ tracer at 23 d after sowing (DAS), imaged by MRI and used as the initial condition for the model. (B) Tracer concentration distribution measured by MRI at 25 DAS. (C) Tracer concentration distribution modeled by R-SWMS at 25 DAS. (D) Correlation between the final (25 DAS) experimental (MRI) and simulated (R-SWMS) 3D tracer concentration distributions. (E) Correlation between the final (25 DAS) experimental (MRI) and simulated (R-SWMS) horizontally-averaged concentration distributions, with horizontal and vertical error bars respectively representing the observed and simulated SDs at each depth.

The best root hydraulic parameter set could accurately reproduce the tracer distribution, as suggested qualitatively by the distributions of the simulated and observed data (Fig. 2B, C), which showed more accumulation in the top part of the column, where most roots are located. In general, the model tended to smooth out the concentration patterns with fewer high-concentration spots in the bottom part of the column.

A quantitative comparison can be performed with the help of correlation plots between the experimental (MRI) and the simulated (R-SWMS) Gd-DTPA^2−^ 3D concentration distributions (Fig. 2D) or 1D concentration profiles (Fig. 2E). In general, it could be seen that the spreading of the differences between measured and simulated concentrations increased with tracer concentration. The optimal parameter set resulted in a RMSE of 1.75 mmol l^−1^ and *r*^2^=0.55 with a slope of 0.66 when a 3D voxel-per-voxel comparison was performed. These values might appear poor, but it must be kept in mind that the spatial resolution was high (0.5 cm voxel width) and that uncertainty existed on the exact location of the root segments in the soil (due to the manual root reconstruction, and hence hardly quantifiable) and the lack of spatial resolution for very fine roots (<200 µm).

**Fig. 3.**
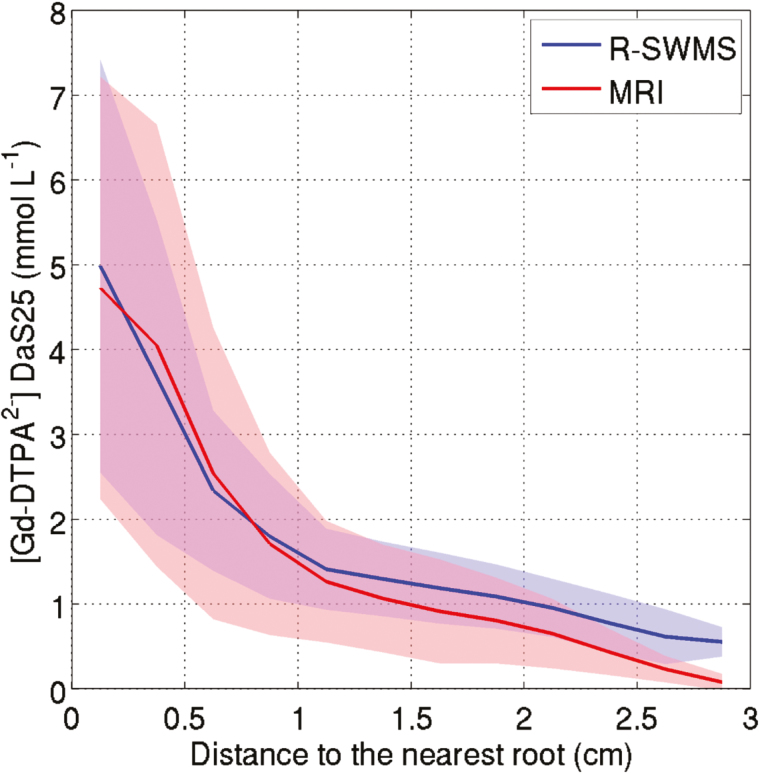
Concentration of Gd-DTPA^2−^ in a voxel versus distance to the nearest lateral root at 25 d after sowing (DAS) as determined experimentally by MRI or simulated using R-SWMS. The voxels were binned with an interval of 0.25 cm. The lines represent the mean concentrations and the shaded areas cover ±1 SD.

This lack of accuracy in the co-registration of the soil and the root system was confirmed by the fact that the RMSE and *r*^2^ were improved to 0.22 mmol l^−1^ and 0.98, respectively, when the tracer concentration was averaged horizontally (Fig. 2E). In this case, the slope of the linear regression was close to unity, indicating that the 1D concentration profiles were almost perfectly reproduced by the model. The good agreement between the experimental and simulated Gd-DTPA^2−^ concentrations at 25 DAS is emphasized in Fig. 3. Indeed, it can be seen that the relationship between the mean tracer concentration and the distance to the nearest lateral root was similar in both cases. Moreover, the standard deviation of both experimental and simulated data were in the same range.

### Sensitivity of simulated tracer distributions to root hydraulic properties

**Fig. 4.**
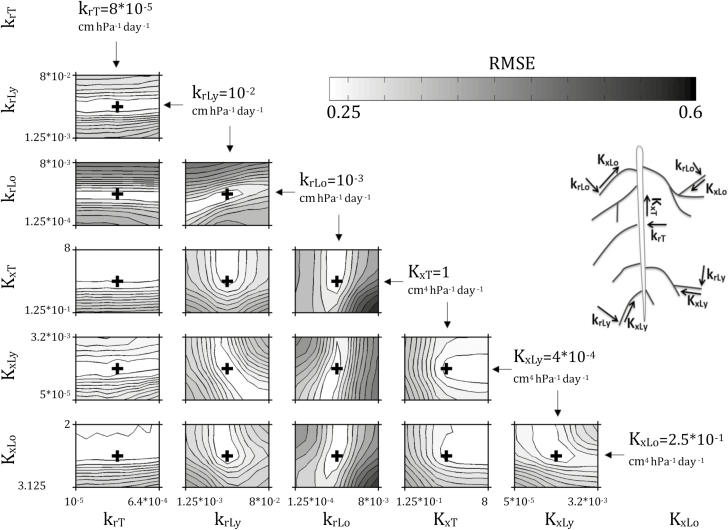
RMSE (mmol l^−1^) between observed and simulated Gd-DTPA^2−^ concentrations at 25 d after sowing (DAS) as a function of different values of the root hydraulic properties considered in the simulations (see Table 1). The lower the RMSE (i.e. lighter shading), the closer the simulation results are to the experimental ones. The black crosses correspond to the lowest RMSE, and to the optimal parameter sets. The corresponding optimal parameters are given for each plot. *K*_x_ and *k*_r_ are in cm^4^ hPa^−1^ d^−1^ and cm hPa^−1^ d^−1^, respectively. The subscripts T, Ly, and Lo refer to taproot, young laterals, and old laterals, respectively.

The response surfaces (logarithm of the RMSE between observed and simulated Gd-DTPA^2−^ concentrations at 25 DAS, eqn 2) around the global optimal root hydraulic parameter set are shown in Fig. 4. The 3D (voxel-to-voxel comparison) RMSE values in the close vicinity to the model optimum (i.e. when the parameters are disturbed from 1/8 to 8 times the optimum) were between 1.75 mmol l^−1^ (the minimal value corresponding to the optimal root hydraulic parameters) and 4.11 mmol l^−1^. These values can be compared to the observed range of Gd-DTPA^2−^ concentrations at 25 DAS, which varied from 0–15.6 mmol l^−1^ with a mean of 2.1 mmol l^−1^. The hydraulic properties of the lateral roots (subscripts Ly and Lo) were much more sensitive parameters than those of the taproot (subscript T). Indeed, the RMSE was completely insensitive to *k*_rT_, because irrespective of its value the taproot did not take up much water and thus did not affect the distribution of solute accumulation. Above a minimal threshold value, *K*_xT_ could be increased without affecting the RMSE value. In other words, there was a minimum value for *K*_xT_ (1 cm^4^ hPa^−1^ d^−1^, its optimum value) above which *K*_xT_ was insensitive, i.e. it was non-limiting for the water fluxes, and thus did not affect water uptake and accumulation of the tracer.

These response surfaces also allowed us to visualize correlations that existed between parameters, and hence trade-offs between model parameters. *k*_rLo_ and *k*_rLy_ were positively correlated (if *k*_rLy_ decreased, then *k*_rLo_ also decreased as well to maintain model performance), *k*_rLy_ and *K*_xLy_ were negatively correlated (an increase/decrease of *k*_rLy_ combined with a decrease/increase of *K*_x,y_ did not influence the modeled concentration), and the same observation could be made for *K*_xLo_ and *K*_xLy_. These correlations between root hydraulic parameters demonstrated how similar distributions of Gd-DTPA^2−^ accumulation around the roots could be obtained from different root hydraulic properties. First, the same distributions of uptake along a root could be obtained when the radial conductivities of young and old segments were simultaneously decreased. On the other hand, to maintain the same uptake from young segments, their axial conductance should increase when their radial conductivity is decreased; however, when the axial conductance of the younger segments is increased, the axial conductance of the older segments should decrease so that the uptake is not shifted towards the younger root segments. These examples show how the correlations between root hydraulic parameters can be related to the hydraulics of single roots (Landsberg and Fowkes, 1978; Meunier *et al.*, 2017). The sensitivity of water uptake density to root hydraulic properties is shown in Supplementary Fig. S2.

The sensitivity analysis indicated that the tracer distribution was more affected by radial conductivities than axial conductances (Fig. 4). This implied that, in our case, *k*_r_ was more limiting than *K*_x_ for water uptake, which is in agreement with the results of Frensch and Steudle (1989). A change in *k*_rLy_ or *k*_rLo_ significantly increased the RMSE. In fact, if *k*_rLo_ becomes higher, it implies that RWU partitioning between young and old root segments will change; old root segments will take up more water and young ones will take less than in the optimal scenario. In the specific case where *k*_rLo_ was multiplied by 6 (6×10^−3^ cm hPa^−1^ d^−1^) and *k*_rLy_ was divided by 2 (5×10^−3^ cm hPa^−1^ d^−1^), all lateral root segments had the same ability to take up water from the soil. The larger RSME for this scenario (Fig. 4) leads us to conclude that the oldest root segments (>5 d-old) should not be able to take up water at a same or higher rate than the youngest ones. This validates our original assumption that *k*_r_ decreases with root maturation (i.e. root ageing). To further demonstrate the impact of the distribution of root hydraulics (and hence root water uptake) on tracer concentration, we also compared the tracer distributions resulting from a scenario in which the taproot was the main location of water uptake with the optimal uptake scenario (see Supplementary Video S1).

### Optimized root hydraulic parameters

**Fig. 5.**
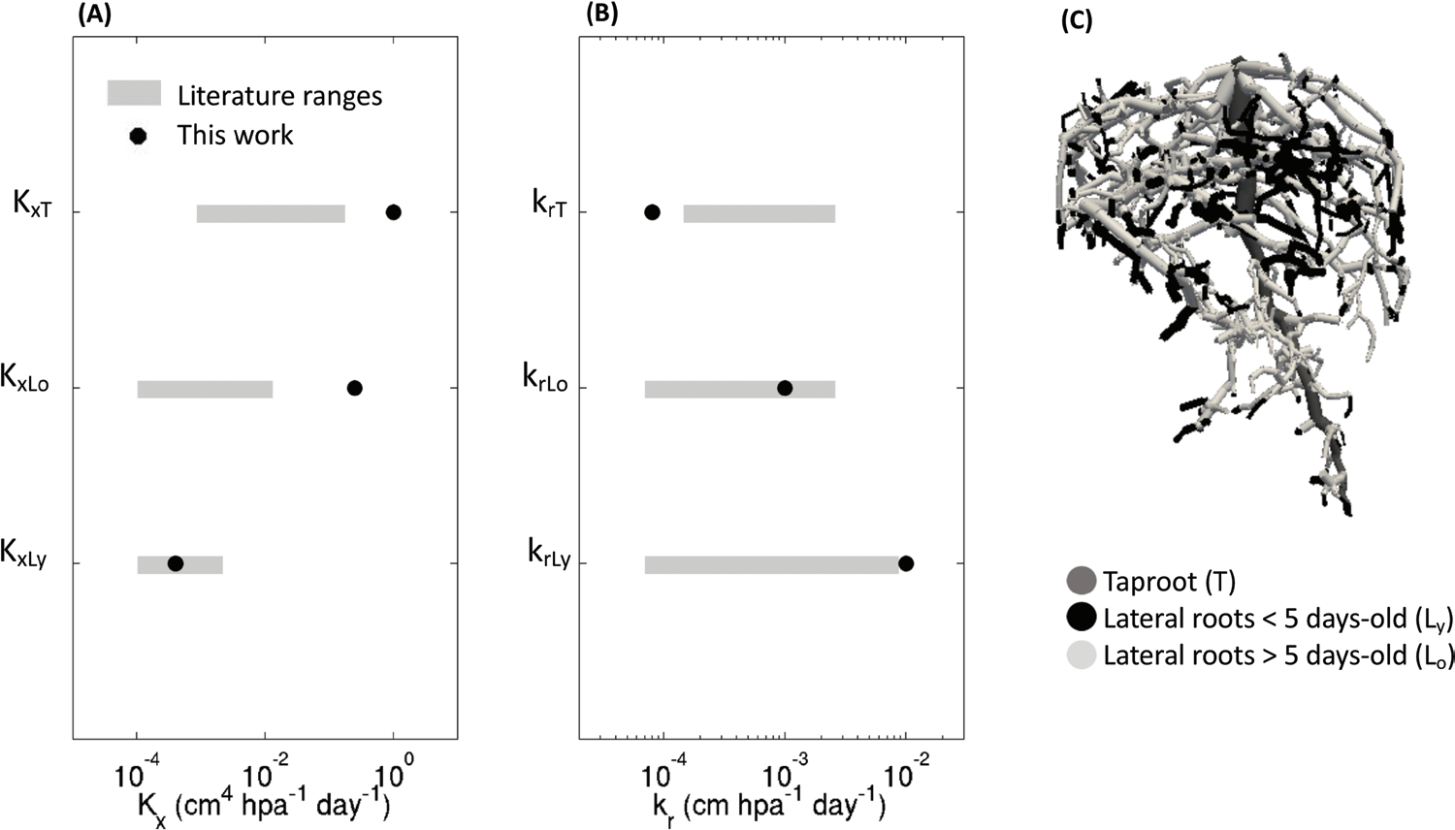
(A, B) Comparison of optimized radial conductivities (*k*_r_) and axial conductances (*K*_x_) determined in the current study with existing literature values for lupin plants. The hydraulic properties of the taproot are indicated by the subscript T, and those of the lateral roots by the subscripts Ly and Lo for young (0–5 d old) and old segments (5–25 d old), respectively. The best root hydraulic properties as determined in the current study are compared with the ranges of values reported by Doussan *et al.* (2006), Bramley *et al.* (2007), Zarebanadkouki *et al.* (2016), and Meunier *et al.* (2018b). (C) Root system architecture at 25 d after sowing.

In Fig. 5, we compare our optimal root hydraulic parameter set with those of Doussan *et al.* (2006), Bramley *et al.* (2007), Zarebanadkouki *et al.* (2016), and Meunier *et al.* (2018b), who all worked on lupins. It can be seen that *K*_x_ is higher in the taproot than in the lateral roots whereas the opposite tendency is observed for *k*_r_. For lateral roots, *K*_x_ increases with root age. As explained earlier, we imposed a decreasing *k*_r_ with root age but *K*_x_ was not subject to any constraint. The maturation of xylem vessels in the early root developmental stages could explain the increase in *K*_*x*_ with age (Vetterlein and Doussan, 2016). The root radial conductivities found in this study spanned two orders of magnitude between the youngest and the oldest root segments. The axial conductances of the different root orders and ages obtained by inverse modeling for the RSAs considered covered four orders of magnitude. It should be noted that the taproot axial conductance (*K*_xT_=1 cm^4^ hPa^−1^ d^−1^) corresponded to the upper limit of the parametric space considered. Moreover, as shown in Fig. 4, the *K*_xT_ value that we found represented the lower limit of conductances that could represent the tracer distribution; however, its value was larger than in previous experiments performed on lupins, and the taproot radial conductivity was smaller. Considering lateral root segments of the same age, our value for axial conductance was close to those reported in the literature for *K*_xLy_ but not for *K*_xLo_. The radial conductivity obtained by optimization for the young (*k*_rLy_) and the old root segments (*k*_rLo_) were in the same ranges as reported in the literature.

An alternative way to assess the reliability of the root hydraulic properties that were determined is to check the root-system total hydraulic conductivity, *k*_rs_ (also referred to *L*_pr_ in the literature), which is the root-system total hydraulic conductance (*K*_rs_) divided by the total root surface area. Bramley *et al.* (2009) measured *k*_*rs*_ as 1.31×10^−7^ m MPa^−1^ s^−1^ for a 14-d-old lupin plant. In comparison, we found *k*_rs_=1.63×10^−6^ m MPa^−1^ s^−1^ for a 25-day-old root system and *k*_rs_=3.8×10^−7^ m MPa^−1^ s^−1^ for a 14-d-old root system, which suggests a good agreement and supports the plausibility of the root hydraulic properties that we determined.

### Interpolations and extrapolations

**Fig. 6.**
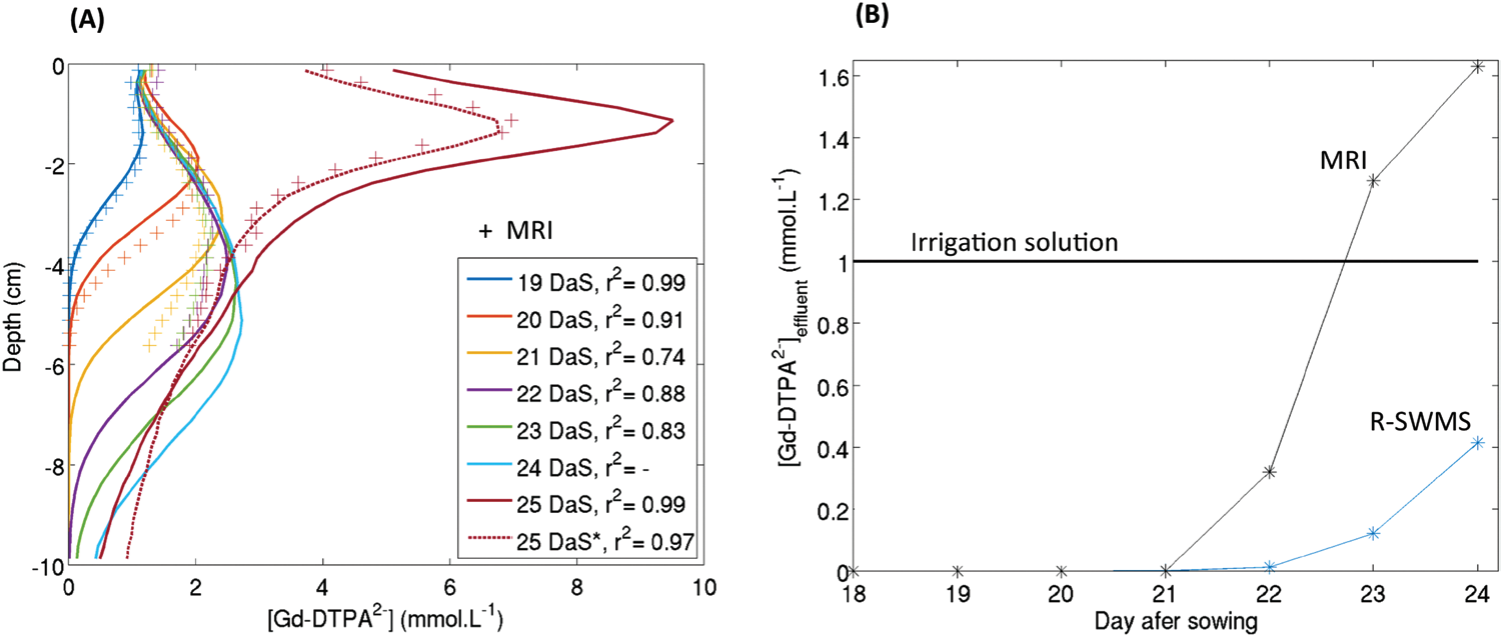
(A): Modeled and measured Gd-DTPA^2−^ concentration profiles. The concentrations were either measured using MRI (crosses) or simulated using the R-SWMS model (lines). No MRI data were available for 24 d after sowing (DAS); the solid lines result from simulating the whole experimental period (18–25 DAS) and the dotted line (25 DAS*) results from simulating 23–25 DAS only. (B) Modeled and measured concentrations of Gd-DTPA^2–^ in the effluent (‘breakthrough curves’). The irrigation solution had a constant concentration over the entire experiment.

In a second step, we used the optimal root hydraulic properties to simulate the whole experiment (from 18–25 DAS). The simulated 1D vertical profiles of Gd-DTPA^2−^ concentrations together with the experimental profiles (MRI) are presented in Fig. 6A. The corresponding determination coefficients were high (covering a range 0.74–0.99), especially for the first two days of the experiment (*r*^2^=0.99 and *r*^2^=0.91, respectively). However, after 2 d, a mismatch between the simulated and observed profiles could be seen, especially for the lower part of the soil column (deeper than 4 cm). At the end of the experiment (25 DAS), the observed and simulated concentration profiles had the same shape (and hence the correlation was good) but the absolute concentration values were different. This discrepancy was also visible in the simulation of tracer concentration in the effluent (the ‘breakthrough curve’, Fig. 6B), which clearly lagged behind the observed data. This was probably due to some preferential flow paths occurring at depths lower than 4 cm. Indeed, the simulations showed that the tracer front was slowly moving downwards (i.e. the front was flat) whereas the MRI-derived Gd-DTPA^2−^ front reached the bottom of the soil column much more quickly in the experiment. The difference in the tracer concentrations at 25 DAS was due to a higher tracer mass in the simulated soil column than in the experimental one. Indeed, since the tracer particles reached the bottom of the column later, there was less tracer mass in the effluent and more left in the soil column. Further optimization of the soil conductivity parameters for that part of the soil profile (e.g. taking into account soil heterogeneities) could have improved the fitting, but was beyond the scope of this study. Despite the fact that the observed breakthrough curve could not be properly modeled when starting the simulation at 18 DAS, we assumed that the root hydraulic parameters were correct as (i) they were very sensitive to the second step of the experiment and not to the first part, (ii) this preferential flow was related to soil parameterization rather than to root properties, and (iii) the impact of the preferential flow on the mass balance of the tracer in the soil profile was accounted for by setting the initial conditions of the concentrations at the last moment of the leaching phase for which a concentration map was available (at 23 DAS; the end of the leaching phase was at 24 DAS) based on the MRI-measured concentration distributions (see 25 DAS* in Fig. 6A).

### Determining RWU distributions

**Fig. 7.**
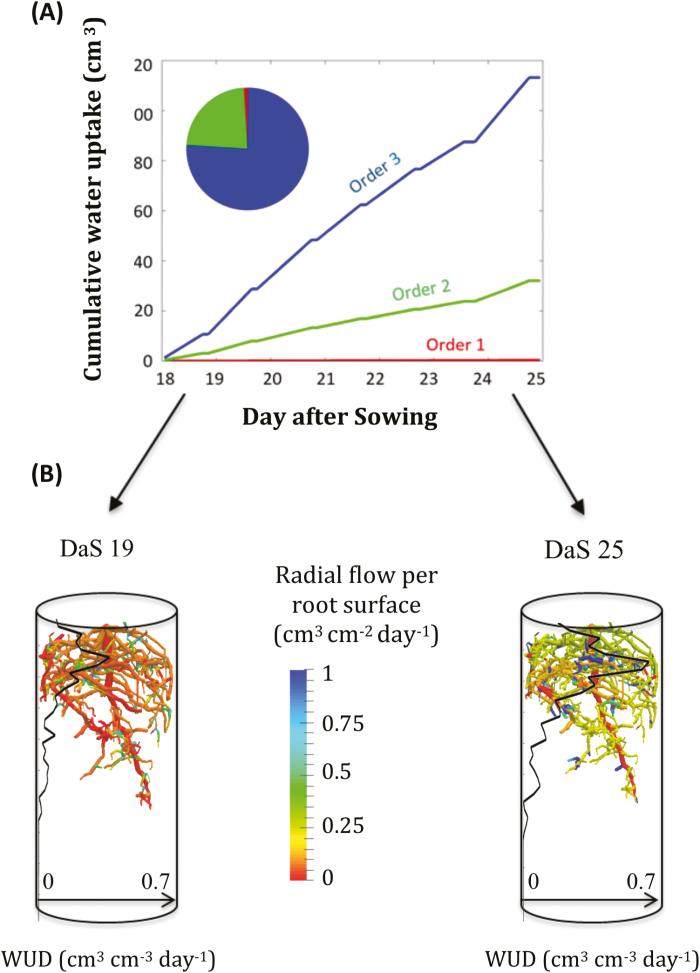
(A) Cumulative water uptake volume by different root orders during the course of the experiment, and pie-chart of the contributions by the different root orders at 25 d after sowing (DAS). (B) Radial root flow distributions over a day during the first (left, 19 DAS) and the last (right, 25 DAS) day of the experiment. The water uptake density (WUD) 1D profiles are also given (black lines).

The optimal parameter set could be used to determine variables of the SPAC that were not directly observable, such as RWU distribution. The total cumulative water uptake over the whole experiment (18–25 DAS) is shown in Fig. 7. Water was mainly taken up by lateral roots (76% by 3rd-order root segments, 23% by 2nd-order, and barely 1% by the taproot). This is in agreement with Zarebanadkouki *et al.* (2016), who also found out that soil water was mainly taken up by lateral roots in a lupin. We also observed (i) an increase in the uptake rate between the first and the last day of the experiment, which was a consequence of the doubling of the transpiration rate during this period (Supplementary Table S1), (ii) that water was mostly taken up close to the root tip, corresponding to a zone with young root segments with high radial conductivity and a reasonable axial conductance, and (iii) that water was taken up over the whole rooting depth both under relatively wet (19 DAS) and dry (25 DAS) conditions. This indicated that soil hydraulic conductivity was not limiting RWU, even under the drier conditions. The mean water contents at 19 DAS and 25 DAS were *θ*_19_=0.36 and *θ*_25_=0.21, and the mean soil water potentials were *h*_19_=–1.86×10^−3^ MPa and *h*_25_=–3.53×10^−3^ MPa (Supplementary Fig. S3).

**Fig. 8.**
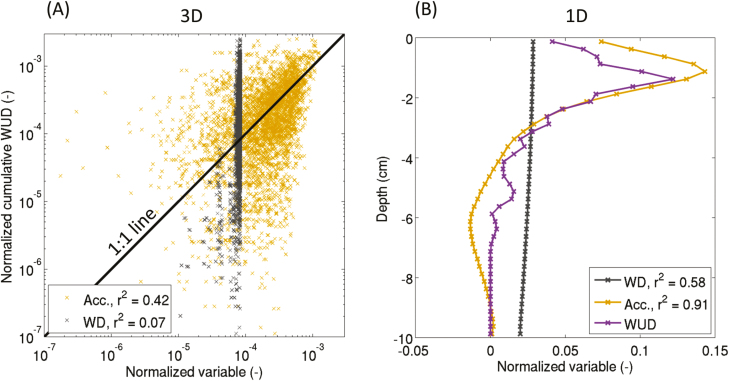
(A) Correlations between 3D water depletion (WD, cm^3^ cm^−3^), 3D Gd-DTPA^2−^ accumulation (Acc., mmol l^−1^), and 3D cumulative water uptake density (WUD) for a voxel size of 0.5 cm (cm^3^ cm^−3^ d^−1^). (B) 1D vertical profiles of WD, Acc., and WUD over one day (24–25 d after sowing, DAS). WD represents the change of water content, Acc. represents the increase of tracer concentration, and the cumulative WUD is the total amount of water taken up by roots in each voxel. The correlations were analysed during the non-leaching period only (24–25 DAS).

The relationships between two proxies, namely water depletion and tracer accumulation, and the WUD during the non-leaching period are shown in Fig. 8. It is obvious that water depletion did not appropriately reflect the cumulative WUD. Gd-DTPA^2−^ accumulation gave an accurate view of the cumulative WUD in 1D (*r*^2^=0.91); however, its accuracy in predicting 3D cumulative WUD distribution was less evident and highly dependent on the spatial resolution that was being considered. The proxies shown in Fig. 8 are the results averaged to a mean voxel size of 0.5 cm.

## Discussion

### Tracer accumulation

We assessed the potential of an experiment involving a plant-excluded tracer to obtain quantitative information on root hydraulics by using a process-based model of the soil–root continuum to fit the 3D concentration build-up around roots during a period with no irrigation. Simulated and observed high concentrations were located similarly, but their actual values could differ. A possible explanation might be that, for high concentrations (>5 mmol l^−1^), the uncertainty range of the MRI-measured concentrations becomes higher (see fig. 7 in Haber-Pohlmeier *et al.*, (2017)).

### Proxies for WUD

**Fig. 9.**
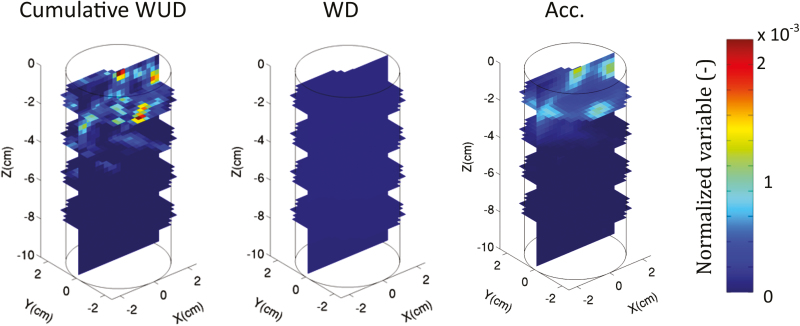
3D distribution of cumulative water uptake density (WUD, cm^3^ cm^−3^ d^−1^), water depletion (WD, cm^3^ cm^−3^), and Gd-DTPA^2−^ accumulation (Acc., mmol l^−1^) for the optimal root hydraulic properties. WD represents the variation of water content, Acc. represents the increase of tracer concentration. All data are from the non-leaching period only (24–25 d after sowing).

The poor correlation between water depletion (WD) and water uptake density (WUD) in the model simulations supports the rejection of the former as a suitable proxy for WUD. During the non-leaching phase, the 3D local tracer accumulation was a much better proxy for WUD than WD, but there was considerable noise in the relationship between local WUD and tracer accumulation, and this noise increased with increasing spatial resolution. Gd-DTPA^2−^ accumulation seemed to be influenced not only by the local cumulative WUD but also by the neighbouring concentrations (or neighbouring uptake) (Fig. 9). For example, a voxel that is subject to a small WUD and that is located next to a voxel in which water is taken up more intensely may experience a significant accumulation of Gd-DTPA^2−^ since the tracer was distributed exponentially around the water-uptake sink (Fig. 3). Moreover, it should be kept in mind that Gd-DTPA^2−^ redistribution took place only during one day after the leaching phase; better correlations could be expected with longer redistribution periods.

### Validation of a FSRSM

The fact that the FSRSM was able to reproduce the observed tracer distribution when the root hydraulic parameters were calibrated could be considered as an indirect validation. Indeed, the values of the optimized parameters were in agreement with what was expected based on root anatomy and they were in accordance with values from previous studies of the same species. We have demonstrated how a calibrated and validated FSRSM could be applied to assess water uptake by different root orders and at different locations along a single root.

### Opportunities

Selection of drought-tolerant genotypes is often based on structural traits such as rooting depth, root-length density, and RSA (de Dorlodot *et al.*, 2007; Trachsel *et al.*, 2010; Paez-Garcia *et al.*, 2015), but local root hydraulic properties are also crucial (Vadez, 2014).

A validated FSRSM can be used to quantify the impact of a combination of root structural and functional traits on root water uptake (Meunier *et al.*, 2017). Coupled with a whole-plant model, a FSRSM allows us to determine ideotypes for RWU (Leitner *et al.*, 2014; van Eeuwijk *et al.*, 2019).

The use of FSRSMs in inverse modeling schemes opens new avenues for translating information obtained from sophisticated experimental tracer methods into information that can be used for practical applications (e.g. obtaining root hydraulic properties *in situ*). Although our study has demonstrated the unique capability of MRI to image root architectures and tracer distributions, there is still a long way to go before it can be used as a standardized and high-throughput method for root hydraulic phenotyping.

Another application of FSRSM is to obtain root hydraulic characteristics using methods based on the isotopic composition of water (e.g. Meunier *et al.*, 2018a).

## Conclusions

This study is the first that combines 4D (space and time) RSA with spatially resolved measurements of root-zone tracer concentrations to validate/parameterize a FSRSM. We have shown that R-SWMS, a FSRSM, can properly represent water and solute fluxes in the root zone. Moreover, 3D tracer distribution maps were demonstrated to contain valuable information for inferring the hydraulic parameters of roots of different orders and ages. The parameter set obtained was in the range of other previous studies of lupin plants. The use of the model also allowed us to unravel the RWU dynamics *in situ*. RWU was shown to be affected by root growth and, in particular, by the root-age distribution, which affected the hydraulic architecture. The 3^rd^-order roots, which represented 70% of the total root length, extracted 76% of the water. The simulations highlighted the fact that changes water content or tracer accumulation were not suitable proxies for water uptake. The validation of models such as the one presented here opens new opportunities for developing drought-tolerant ideotypes.

## Supplementary data

Supplementary data are available at *JXB* online.

Table S1. Cumulated water volumes during the experiment. 

Table S2. van Genuchten–Mualem soil hydraulic parameters for the FH31-soil used in the simulations. 

Fig. S1. Timeline showing the experimental protocol together with the timing of irrigation and MRI scanning. 

Fig. S2. Sensitivity analysis using RMSE between the simulated WUD at 25 DAS for optimal and adjusted parameters as a function of root hydraulic property values considered in the simulations.

Fig. S3. Water retention curve based on the van-Genuchten–Mualem parameters used in the simulation. 

Movie S1. Dynamics of tracer distribution through the soil column for two different root hydraulic architectures, namely optimal parameters and taproot only (see Supplementary data file for full details). 

Supplementary Figures S1-S3 and Tables S1-S2

Supplementary Video S1

## Abbreviations:

2D/3D/4D: Two/three/four-dimensional
DAS: days after sowing
FSRSM: functional–structural root-system model
FOV: field of view
Gd-DTPA2−: gadolinium diethylene-triamine-penta-acetate
MRI: magnetic resonance imaging
neutron CT: neuton computed tomography
RMSE: root mean-square of error
RSA: root-system architecture
RWU: root water uptake
SPAC: soil–plant–atmosphere continuum
X-ray CT: X-ray computed tomography
