# Supplementary material for: Functional–structural root-system model validation using a soil MRI experiment
Source: J Exp Bot. 2019 Feb 19;70(10):2797–809. doi: 10.1093/jxb/erz060 (PMC6509106; doi:10.1093/jxb/erz060)
Supplement: Supplementary Figures S1-S3 and Tables S1-S2 [file erz060_suppl_supplementary_figures_s1-s3_tables_s1-s2.pdf]

Supplementary sections

Table S1. Cumulated water volumes during the experiment, adapted from (Haber-Pohlmeier et al. 2017), Table 1

Day after Sowing	Cumulative transpired volume (cm^3)	Cumulative irrigated volume (cm^3)	Cumulative effluent volume (cm^3)	Average water content (cm^3cm^{-3})
18	0	0	0	0.35
19	13.7	22.1	7.2	0.36
20	32.9	39.6	8.8	0.34
21	55.0	69.4	15.6	0.34
22	70.3	93.7	23.4	0.35
23	86.1	115.5	29.4	0.35
24	99.5	136.2	35.3	0.36
25	131.2	136.2	35.3	0.20

During the irrigation phase of the experiment, the amount of irrigation solution as well as the effluent were measured gravimetrically; the daily transpiration volume was then inferred by mass balance assuming that the water storage in the column did not change. At the end of the experiment, when the irrigation was stopped, the transpiration volume was derived from the change in weight of the column and the measured effluent. The cumulated transpired volume after one day was modified from 16.7 to 13.7 after discussion with the authors. The average water content values were then re-calculated as follows (initial water content is 0.35): first the change in water volume was determined (=Irrigation-Transpiration-Effluent), then the total water volume was estimated by adding the change in water volume to the total water volume of the previous day, finally the average water content was obtained by computing the ratio between the total water volume and the column volume.

Table S2. van Genuchten – Mualem soil hydraulic parameters for FH31-soil used in the simulations (Schröder 2014)

θ_r ($cm^3\ cm^{-3}$)	θ_s ($cm^3\ cm^{-3}$)	a (cm^{-1})	n (-)	K_s ($cm\ d^{-1}$)	L (-)
0.005	0.397	0.03	3.6	647	0.5

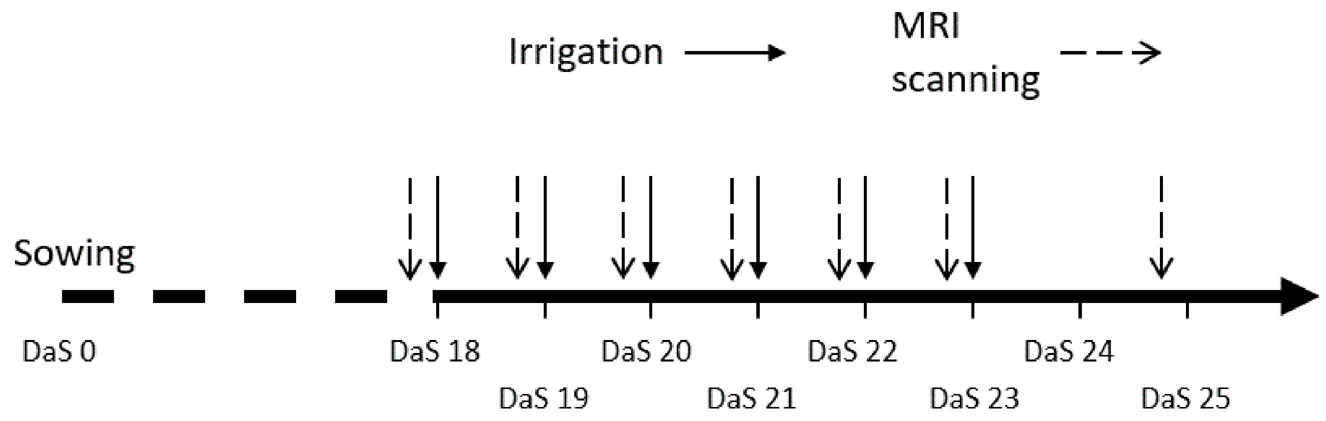

Figure S1. Timeline showing the experimental protocol together with the irrigation (continuous arrow) and the MRI scanning (dashed arrow) timing.

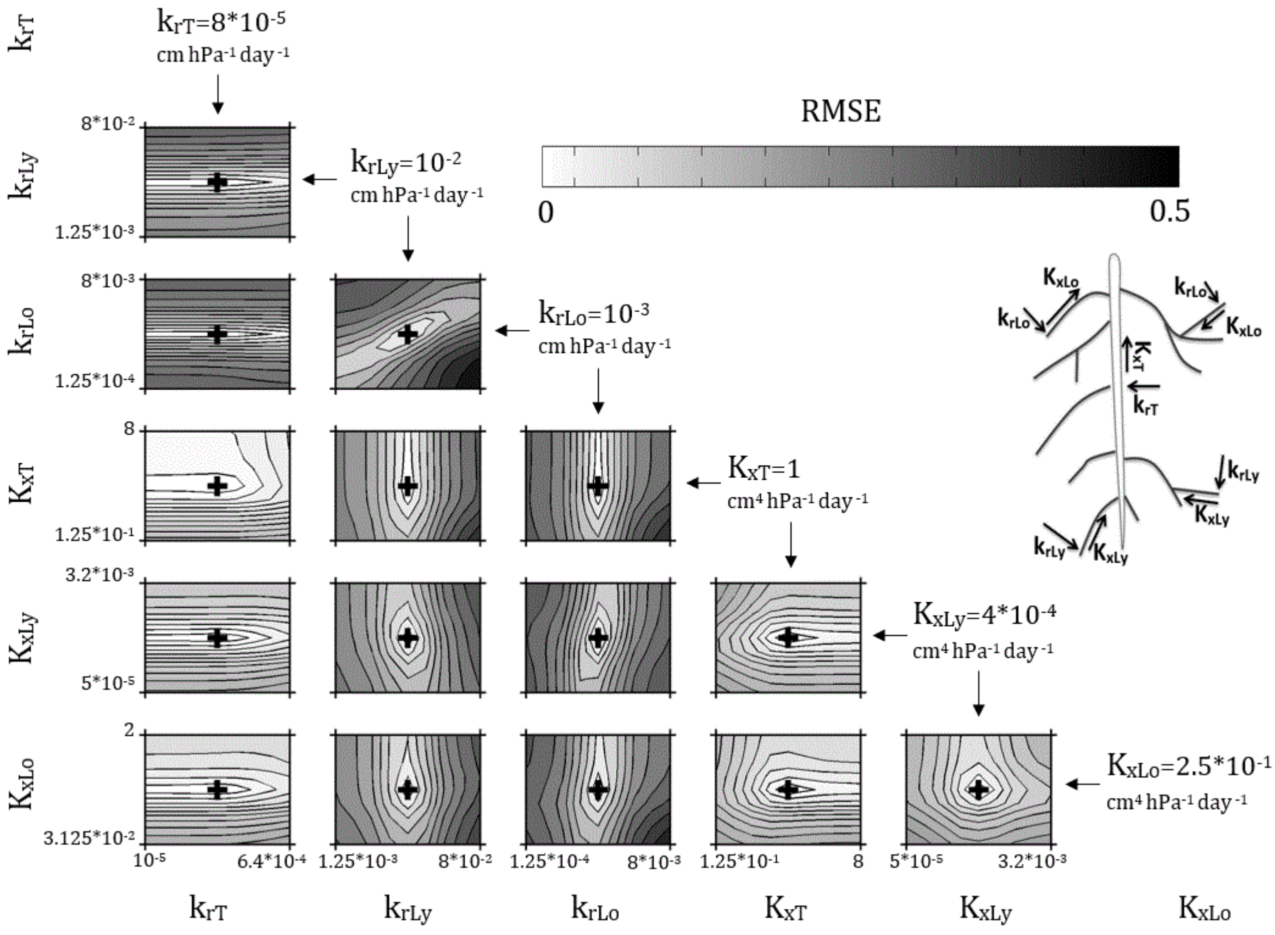

Figure S2. RMSE (cm^3 cm^{-3} day^{-1}) between the simulated WUD at 25 DaS for the optimal and the perturbed parameter as a function of root hydraulic property values considered in the simulations. The lower the RMSE (i.e. the lighter in the figure), the closer the simulation results to the optimal ones (i.e. simulated with optimal parameter set). The black crosses correspond to the lowest RMSE, and to the optimal parameter sets. The corresponding optimal parameters are given for each plot. K_x and k_r are in cm^4 hPa^{-1} day^{-1} and cm hPa^{-1} day^{-1}, respectively. The subscripts T, Ly and Lo refer to taproot, young laterals and old laterals, respectively.

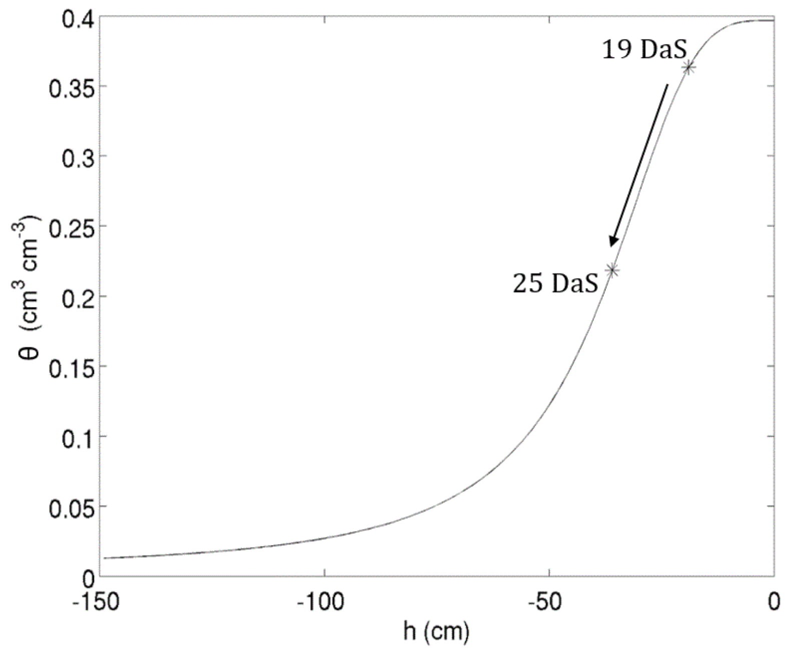

Figure S3. Water retention curve based on the van-Genuchten-Mualem parameters used in the simulation (see parameters of Table S2). The two stars represent the soil water status at DaS 19 ($\theta = 0.36$) and 25 ($\theta = 0.21$).

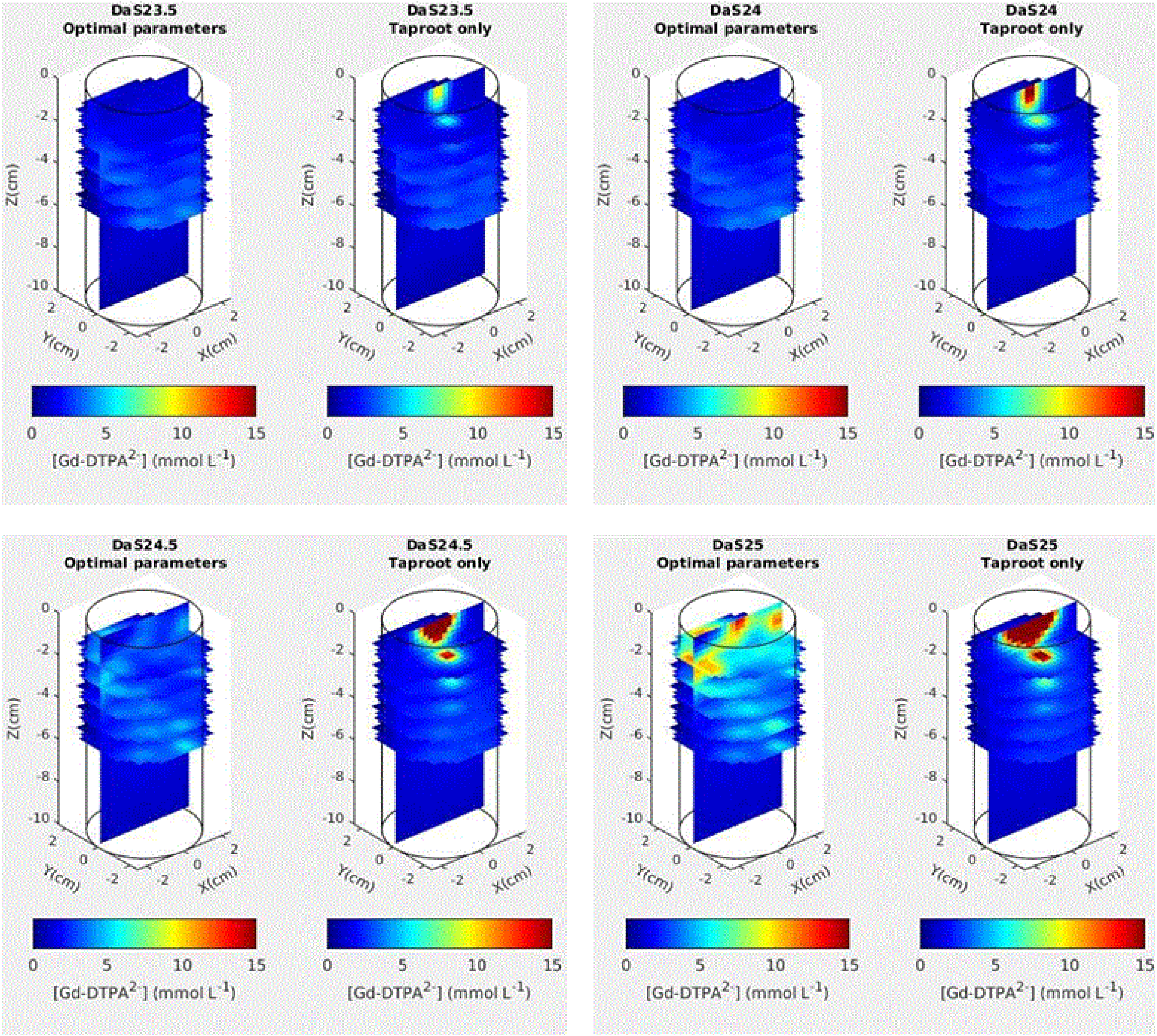

Movie S1. Dynamic of tracer distribution through the soil column for two different root hydraulic architectures (Optimal parameters and Taproot only). The set of root hydraulic parameters considered are K_{xT}=1 cm^4 hPa^{-1} day^{-1}, K_{xLy}=4*10^{-4} cm^4 hPa^{-1} day^{-1}, K_{xLo}=2.5*10^{-1} cm^4 hPa^{-1} day^{-1}, k_{rT}=8*10^{-5} cm hPa^{-1} day^{-1}, k_{rLy}=10^{-2} cm hPa^{-1} day^{-1} and k_{rLo}=10^{-3} cm hPa^{-1} day^{-1} for the scenario "Optimal parameters" and K_{xT}=1 cm^4 hPa^{-1} day^{-1}, K_{xLy}=10^{-4} cm^4 hPa^{-1} day^{-1}, K_{xLo}=10^{-4} cm^4 hPa^{-1} day^{-1}, k_{rT}=1 cm hPa^{-1} day^{-1}, k_{rLy}=10^{-5} cm hPa^{-1} day^{-1} hPa^{-1} and k_{rLo}=10^{-5} cm hPa^{-1} day^{-1} for the scenario "Taproot only".
